# Hepatocellular Carcinoma: Past and Future of Molecular Target Therapy

**DOI:** 10.3390/diseases4010001

**Published:** 2015-12-25

**Authors:** Khanh Nguyen, Kerri Jack, Weijing Sun

**Affiliations:** University of Pittsburgh Medical Center, University of Pittsburgh Cancer Institute, University of Pittsburgh School of Medicine, 5150 Center Ave. 5th floor, Pittsburgh, PA 15232, USA; nguyenkt5@upmc.edu (K.N.); Jackk@upmc.edu (K.J.)

**Keywords:** hepatocellular carcinoma, HCC, molecular therapy, targeted therapy, immunotherapy, sorafenib

## Abstract

Hepatocellular carcinoma (HCC) is one of the most common causes of cancer related mortality worldwide. The incidence of HCC has been increasing annually. Viral infection, alcohol usage, and other causes of cirrhosis have been identified as major risk factors for HCC development. The underlying pathogenesis has not been as well defined. There have been multiple hypotheses to the specific mechanisms of hepatocarcinogenesis and they share the common theme of chronic inflammation, increase oxidative stress, and genomic alteration. Therapeutic options of HCC have been primarily local and/or regional including transplantation, resection, and radial frequency ablation, chemoembolization or radio-embolization. For unresectable or metastatic disease, the options are limited. Conventional chemotherapeutic options have been noted to have limited benefit. Sorafenib has been the one and only systemic therapy which has demonstrated modest overall survival benefit. This has led to more extensive research with focus on targeted therapy. Numerous pre-clinical and early phase clinical studies have been noted but failed to show efficacy in later phase clinical trials. In an effort to identify new potential therapeutic options, new understanding of underlying pathways to hepatocarcinogenesis should be one of the main focuses. This leads to development of more molecularly targeted agents to specific pathways, and immunotherapy. This article provides a review of major studies of molecular targeted agents which attempts to target these specific pathways in HCC.

## 1. Introduction

Hepatocellular carcinoma (HCC) is the seventh most common cancer in the world and the third in cancer related mortality in adults worldwide [1]. According to the Surveillance Epidemiology and End Results (SEER) registry database, liver cancer remains one of the few cancers which has continued to increase in incidence and at one of the largest annual increase in incidence rates [2]. Usually HCC is diagnosed in later stages and has a median survival of about 20 months after diagnosis [3]. Selected patients with localized disease may be treated with curative intents with resection, liver transplantation, or local therapy [4]. However, the majority of patients with HCC are not candidates for resection [4]. The five year survival of patient who undergo surgical resection is about 42%–67% [5]. Liver transplantation may be a potential therapeutic option for those patients who are not candidates for resection but still have localized disease. Liver transplantation in HCC have been shown to have a five year survival up to 75% [6,7,8]. For patients who do not meet criteria for resection or transplantation, nonsurgical local-regional therapies such as transarterial chemoembolization (TACE), radioembolization, cryoablation, and radioablation could be effective but with as high as 80% recurrence and limited to mostly patient without extrahepatic disease or severe liver dysfunction [9,10]. System therapy with chemotherapy has not shown significant effect on survival. For a while, doxorubicin was considered as an agent of choice for HCC, however, those data were from small studies or analyses in the earlier decades [11]. Most studies with cytotoxic chemotherapy have only showed moderate activity.

Due to the aggressive nature of this disease, especially in the advanced setting, there has been more focus on molecularly targeted therapies. So far, sorafenib is the first and only target oriented agent approved to have survival benefits in systemic therapy of HCC. Immunotherapy with checkpoint inhibition has revealed very encouraging data in HCC therapy recently.

## 2. Epidemiology

According to the SEER database from 2006 to 2010, the age-adjusted HCC incidence was 5.9 per 100,000 persons and mortality from liver cancer was 4.3 per 100,000 persons in the United States [12]. The incidence of HCC in men is three times more than in females [13]. Asians and Pacific Islanders have the highest incidence and mortality at 11.7 per 100,000 persons and 8.2 per 100,000 persons respectively [12]. Risk factors contributing to the development of HCC are commonly due to chronic infection with hepatitis B or hepatitis C. Cirrhosis from alcohol liver disease is also an important risk factor. Worldwide, more than half of HCC is attributed to chronic HBV infection and 70%–80% of these cases are in Asian countries which are highly HBV endemic areas [1,14]. In developed countries such as the United States and European countries, the incidence is low and usually acquired in adulthood [13]. In these HBV endemic areas, there is usually an association with aflatoxin exposure which has a synergistic effect in increasing the risk of HCC development [15]. Other common risk factors include chronic alcohol consumption, and conditions leading to cirrhosis including hemochromatosis and alpha-1-antitrypsin [16,17,18]. In recent years, increased incidences of HCC secondary to metabolic abnormality (e.g., nonalcoholic steatohepatitis, NASH) have been reported [19,20].

## 3. Pathogenesis

Although HCC is one of the most common tumors in the world, the underlying pathogenesis of HCC remains poorly understood. The reason behind this may be that the pathogenesis behind hepatocarcinogenesis is multifactorial. In patients with hepatitis B virus (HBV) and hepatitis C virus (HCV) infections, the immune system elicits an increased T-cell response to fight off the viral infections. The T-cell response would trigger sustained inflammation, liver injury, and repair cycle which increases the chance of propagation of genetic alterations leading to carcinogenesis [16,21]. These viral infections will increase ER stress inducing oxidative stress. Under constant oxidative stress, there are increased generation of free radicals leading to genetic alterations and activation of liver stellate cells, which is associated with cellular proliferation and hepatic fibrosis [16,22]. As with viral infection, chronic alcohol intake has been noted to increase pro-inflammatory cytokines and lead to chronic hepatocyte destruction and regeneration cycle resulting in cirrhosis. A chronic alcohol intake also increases the oxidative-stress mechanisms contributing to fibrosis and cirrhosis. Ingestion of aflatoxin B1, fungal toxin, has been associated with the development of HCC through a specific p53 mutation. The p53 tumor suppressor plays an important role in repair of genomic alterations and cellular apoptosis. Inactivation or mutations of the p53 tumor suppressor facilitate the proliferation of hepatocytes in the face of genomic instability leading to HCC [16,23]. These mechanisms suggest some common theme to hepatocarcinogenesis pathway including p53 inactivation or mutation, chronic inflammation leading to persistent hepatocyte necrosis and regeneration causing genetic alteration, and oxidative stress leading to mutagen and fibrosis which can lead to more genetic alteration [16]. Independent of viral hepatitis, alcohol or toxins as etiologies of HCC, there are increasing incidences of nonalcoholic fatty liver disease (NAFLD) and nonalcoholic steatohepatitis (NASH) resulting in HCC. NAFLD/NASH usually develops in association with metabolic syndrome (hypertension, type 2 diabetes mellitus, dyslipidemia). The exact mechanism which leads NASH transformation into HCC is unclear but it has been suggested being related to insulin resistance—causing hyperinsulinemia which drives adipose tissue accumulation and cell proliferation. This subsequently increases toxic lipid metabolites triggering constant inflammatory response through increase cytokines such as interleukin-6 and tumor necrosis factor and hepatocyte proliferation through factors such as insulin growth factor-1 (IGF-1). The upregulation of IGF-1 pathway has been noted to contribute to hepatocarcinogenesis [19,20].

## 4. Systemic Chemotherapy

Generally, cytotoxic systemic chemotherapy has had modest efficacy with short median survival of less than a year [24]. There is no established standard cytotoxic chemotherapy regimen in HCC. Treatments usually have been limited due to this patient population which has significant comorbidities and liver dysfunction [24]. Doxorubicin is the agent relatively evaluated the most, which has only shown 8%–24% overall response rate (ORR) with overall survival (OS) 10 weeks to 6 months [25,26,27,28,29]. 

Fluoropyrimidines base have been evaluated by multiple studies. In combination with leucovorin, 5-fluorouracil have been shown to have ORR between 7%–28% with median OS around 3.8 months and median time to progression (TTP) of 2.4 to 5.7 months [30,31]. The combination of 5-fluorouracil with oxaliplatin was studied in Asia with 371 patients of advanced HCC and showed a median ORR of 8%, median progression free survival (PFS) was 2.93, and median OS was 6.4 months [32]. Capecitabine, an oral fluoropyrimidine, was studied in multiple trials and showed ORR of 3%–11% with median PFS of 3–6 months and median OS of 5–15 months [33,34,35]. The use of capecitabine with oxaliplatin (XELOX) was also evaluated and showed an ORR of 6% with median PFS of 4 months [36].

In addition to fluoropyrimidines, gemcitabine and cisplatin have been evaluated extensively. Single agent gemcitabine has an ORR of about 5% with median OS of 6.9% [37,38]. When gemcitabine is used in combination with oxaliplatin (GEMOX), the data seems promising with the ORR around 18%–22% andthe median OS of 4–11 months [39,40,41]. Gemcitabine and cisplatin have also been studied together and showed an ORR of 9%–20% and median OS of 18–21 weeks [42,43].

## 5. Molecularly Targeted Agent

### 5.1. Angiogenesis

HCC is hypervascular in nature and is supplied by the hepatic arteries as opposed to the portal vein for normal liver parenchyma [44,45]. Because of the hypervascular nature of HCC, angiogenesis, the process of forming new blood vessels from previous vessels, plays a vital role in its pathogenesis and therefore, one of the major targets for therapy. Angiogenesis is vital for tumor growth and metastasis. Angiogenesis in HCC is based on the activation and proliferation of endothelial cells. Activated endothelial cells break down extracellular matrix and basement membrane which result in release of angiogenic factors which includes vascular endothelial growth factor (VEGF), basic fibroblast growth factors (bFGF), platelet-derived growth factor (PDGF), and transforming growth factor-beta (TGFβ) [45]. These factors in turn activate endothelial cells through receptor tyrosine kinases (RTK) and the RAS/RAF/MEK/ERK pathway and PI3K pathway which leads to proliferation and migration of endothelial cells to form a new tubular structure and lumen for new vessels [45,46]. These new vessels are different from normal vessel in the aspect of leaky vessel, incomplete basal membrane, irregular diameter, and branching pattern [45]. Arterialization and sinusoidal capillarization are characteristic of HCC and are represented in the classic vascular enhancement pattern on computed tomography for diagnosis of HCC [47].

VEGF has been shown to have a poor prognosis in HCC when overexpressed [48]. To inhibit angiogenesis, numerous studies have focused on targeting VEGF and VEGF receptor (Table 1). Approximately one third of molecularly directed therapies in clinical evaluation are directed at VEGF or its receptor [49]. Of all the molecular therapeutics for HCC, the most effective agent is sorafenib.

**Sorafenib**: Sorafenib is a small molecule serine/threonine/tyrosine kinase inhibitor which inhibits BRaf, c-Raf, VEGFR1, VEGFR2, VEGFR2, PDGFR-α, PDGFR-β, RET, FLT-3, and c-kit [46,50,51]. It is the first agent to show an overall survival benefit in advanced HCC [50]. There were two major trials which were pivotal to the regulatory approval of the therapy globally and represented a breakthrough in management of advanced HCC. In the multicenter, double-blind, phase III Sorafenib HCC Assessment Randomized Protocol (SHARP) study, 602 patients with advanced HCC who had not previously received systemic therapy were randomized to receive oral sorafenib or placebo [52]. The SHARP study showed a significant median overall survival (OS) benefit for patients with advanced HCC from 7.9 months in the placebo arm to 10.7 months in the sorafenib arm with hazard ratio (HR) of 0.69%; 95% confidence interval (CI) of 0.55–0.87; *p* = 0.001 [52]. In the phase III Asia-Pacific region study, the median OS was noted to be 6.5 months in the sorafenib arm *vs.* 4.2 months in the placebo arm (HR, 0.68; 95% CI, 0.50–0.93; *p* = 0.014) [53]. The toxicities seen in these two studies were very similar with increased rates of diarrhea, weight loss, hand-foot skin reaction, and electrolyte abnormalities (hypophosphatemia) [52,53]. The patient population in these studies was noted to contain 97% Child-Pugh class A patients, and over 90% with ECOG performance status of 0 or 1 [52,53]. Interestingly, the patients in these two studies showed only 2% and 5% overall response rate in the sorafenib arm [52,53]. The use of sorafenib in combination with cytotoxic agent is also being evaluated. Abou-Alfa and colleagues conducted a multinational phase II randomized, double-blind study of doxorubicin with sorafenib *vs.* doxorubicin alone in patients with advanced HCC. They randomized 96 patients and demonstrated a significant improvement in the TTP, PFS, and OS in the combination of doxorubicin and sorafenib arm (mTTP of 6.4 months *vs.* 2.8 months, mPFS of 6.0 months *vs.* 2.7 months, and mOS of 13.7 months *vs.* 6.5 months) [54]. Based on the potential advantage of the doxorubicin and sorafenib combination, a phase III study of sorafenib with and without doxorubicin has been conducted (CALGB 80802). Unfortunately, the study was suspended after the second planned interval analysis due to unlikely advantages of the combination with further resulting patients.

The use of sorafenib in combination or following local therapy such as TACE and surgery has also been an area of interest. TACE is a palliative therapeutic option for patients with unresectable HCC with improved survival when compared to conservative treatment. Survival at 1 year, 2 years, and 3 years were 75%, 50%, and 29% for embolization; 82%, 63%, and 29% for chemoembolization; and 63%, 27%, and 17% for control group [55]. It has been noted that TACE-induced injury to the liver will upregulate angiogenic factors including VEGF and PDGFR, leading to tumor angiogenesis [56]. In a phase III study of sorafenib following TACE in 458 patients with unresectable HCC in Japan and South Korea, there was no significant evidence of benefit to sorafenib with a median TTP of 5.4 months *vs.* 3.7 months for the sorafenib *vs.* placebo groups, respectively (HR, 0.87; 95% CI, 0.70–1.09; *p* = 0.252) [57]. There have been multiple phase II studies to evaluate the use of sorafenib with TACE. Park *et al.*, evaluated concurrent TACE with sorafenib in a prospective, single-arm, phase II study with 50 patients with unresectable HCC and reported median TTP of 7.1 months, and 6 month PFS of 52% [58]. In the global SPCE trial, a phase II randomized, double-blind study on sorafenib or placebo in combination with doxorubicin-eluting beads TACE, the authors noted improved TTP with HR of 0.797 but median TTP had only 3 days difference [59]. In the phase II SOCRATES trial which was a multicenter study evaluating TACE plus sorafenib in 43 patients with unresectable HCC, the median TTP was 16.4 months, median OS was 20.1 months, and DCR of 74.4%. The most common adverse events were diarrhea, hand-foot syndrome, fatigue, decreased appetite, and alopecia [60]. The START trial, a phase II prospective single-arm multinational study of sorafenib in combination with doxorubicin-based TACE in patient with unresectable, reported median PFS of 384 days (12.8 months), median TTP of 415 days (13.8 months), and 3-year OS of 86.1% with the most common grade 3 or worse adverse events were hand-foot syndrome and thrombocytopenia. The START trial used an interrupted dosing schedule where sorafenib was held 4 days prior and after TACE [61]. The data from these studies showed some encouraging data, and the concept of sorafenib in combining with TACE is being further evaluated in large phase III setting including ECOG 1208 (US), TACE-2 (UK), and TACTICS (Japan) trials. However, because of heterogeneity of regimens and schedules in local regional therapy, the enrollments of these trials have been an issue. ECOG 1208 was suspended recently because of slow patient reclusion. Hopefully, data from these studies may bring us some knowledge of the concept.

Sorafenib was evaluated in the adjuvant setting in a small, plot phase II study comparing sorafenib after hepatic resection to hepatic resection alone. The results on 31 patients noted time to recurrence in the sorafenib arm was 21.45 months compared to 13.44 months in the control arm (*p* = 0.006) [62]. The recurrence rate was 29.4% *vs.* 70.7% for the sorafenib group *vs.* control arm, respectively (*p* = 0.032) [63]. In another study, Zhang *et al.*, evaluated adjuvant sorafenib following resection of HCC *vs.* control groups in 78 patients. The study reported no difference in recurrence-free survival (11.0 months in the control group *vs.* 11.7 months in the sorafenib group, *p* = 0.702) [64]. The OS was prolonged in the sorafenib *vs.* control group at 32.4 months *vs.* 25.0 months respectively (*p* = 0.046). There was also an increase in the post-recurrence survival at 22.2 *vs.* 4.4 months for the sorafenib *vs.* control groups, respectively (*p* = 0.003). The study did not show any differences in the recurrence rate [63]. Similarly, the phase III STORM study of sorafenib after surgical resection or local ablation in 1114 patients did not meet their primary endpoint of recurrence-free survival or secondary endpoints of time to recurrence and OS [64]. These studies suggest a potential role for sorafenib as an adjuvant therapy and may benefit from further evaluation.

**Sunitinib**: Sunitinib, like sorafenib, is an oral multi-kinase inhibitor which inhibits VEGF-receptors, PDGFR, c-KIT, FLT-3, RET, and CSF-1R. In multiple phase II clinical trials, advanced HCC patients were treated with sunitinib and was found to have an ORR of 2.7%–12% and median OS of 5.8–9.8 months [65,66,67,68]. In the phase II trials with high dose of sunitinib (50 mg daily for four weeks and no therapy for 2 weeks), it was noted to have a high incidence of toxicities with 80% of the patients having grade 3 or 4 toxicity such as thrombocytopenia, neutropenia, asthenia, and hand-foot syndrome [65,68]. The studies also noted about 10.8%–17.6% with fatal treatment-related toxicity [65,68]. In a large phase III trial comparing sunitinib at 37.5 mg daily for four out of every six weeks with sorafenib at 400 mg twice daily. Unfortunately, the results showed that sunitinib was inferior to sorafenib in 1074 patients with advanced HCC and Child-Pugh class A disease. The trial was terminated prematurely due to drug-related toxicity and inferior outcomes. The median OS was 7.9 months in the sunitinib arm and 10.2 months in the sorafenib arm (HR, 1.30; 95% CI 0.99–1.30; *p* = 0.001). The rate of severe adverse effects was noted to be more in the sunitinib arm [69].

**Linifanib**: Linifanib, a novel selective inhibitor of VEGFR and PDGFR tyrosine kinases, have been evaluated as options beyond sorafenib. In preclinical model, linifanib has shown potent antiproliferative and apoptotic properties [70]. In a phase I trial, two HCC patients had stable disease lasting 17 months. Toxicity profile was noted to be comparable to other VEGFR inhibition agents [71]. In a phase II evaluation, linifanib was shown to have an estimated ORR of 9.1%, median OS of 9.7 months, median TTP of 3.7 months, and an acceptable safety profile [72]. Linifanib was evaluated in a phase III as a first-line therapy *vs.* sorafenib in advanced HCC patients who had no prior systemic therapy, however, it did not meet the primary endpoint of the study with median OS of 9.1 months with linifanib and 9.8 months with sorafenib (HR 1.046; 95% CI 0.896–1.221). Although there may be prolonged PFS in the linifanib arm (5.4 months *vs.* 4.0 months), linifanib was less tolerable with more adverse events, serious adverse events, dose reduction, interruptions, and discontinuation [73].

**Brivanib**: Brivanib, a dual kinase inhibitor of VEGFR-2 and fibroblast growth factor receptor (FGFR)-2, has been evaluated as potential therapeutic option for HCC with preclinical study showing significantly suppressed tumor growth in five of six xenograft lines [74]. Phase II clinical trials showed promising results with ORR of 4%–7% and median OS of 9-10 months [75]. Two multicenter phase III trials were conducted to evaluate the use of brivanib in the first-line and second-line setting. In the BRISK-FL (Brivanib Study in Patients at Risk) study, brivanib was compared to sorafenib in the first-line setting. The study showed a median OS of 9.5 months for the brivanib arm and 9.9 months for the sorafenib arm (HR, 1.06; 95% CI, 0.93–1.22; *p* = 0.373) and no difference in the median TTP either (4.2 months for brivanib and 4.1 months for sorafenib (HR, 1.01; 95% CI, 0.88–1.16; *p* = 0.853) [76]. Brivanib was less tolerable with more adverse effects and high rates of discontinuation. The primary end point to achieve a noninferior median OS was not met [76]. In the second-line setting, the BRISK-PS was conducted to evaluate brivanib *vs.* placebo in patients with Child-Pugh class A or B HCC who previously have failed or were intolerant to sorafenib. The study did not reach the primary endpoint of significant difference in median OS (9.4 months in the brivanib arm *vs.* 8.2 months in the placebo arm (HR, 0.89; 95 % CI 0.69–1.15; *p* = 0.3307), but there was an improvement in the median TTP with 4.2 months in the brivanib arm *vs.* 2.7 in the placebo arm (HR, 0.56; 95% CI, 0.42–0.78; *p* = 0.0001) and the objective response rate was 12% in the brivanib arm *vs.* 2% in the placebo (odds ratio, 5.75; 95% CI, 1.40–23.62; *p* = 0.0032) [77]. The most common toxicities associated with the treatment were hypertension (19%), hyponatremia (18%), fatigue (15%) and decreased appetite (12%) [77].

**Cediranib**: Cediranib (AZD2171) is a novel oral tyrosine kinase inhibitor of inhibitor of VEGFR, c-kit, PDGFR-β and FLT-4. Preclinical evaluation of cediranib showed *in vitro* and *in vivo* impairment of proliferation and migration of endothelial cells and angiogenesis [78]. In a phase II study in patients with unresectable or metastatic HCC, the median OS was 5.8 months and median TTP was 2.8 months. The grade 3 or greater toxicity profile showed fatigue (46%), anorexia (25%), hypertension (21%), and elevated alanine aminotransferase (ALT) (18%) [79]. In another phase II clinical study of patients with advanced HCC by Zhu *et al.*, the dosing of cediranib was decrease to 30 mg daily and showed the median PFS was 5.3 months, median OS was 11.7, and an estimated 3 months PFS rate was 77%. The grade 3 or greater toxicities included hypertension (29%), hyponatremia (29%) and hyperbilirubinemia (18%) [80]. These studies did not show significant response to therapy and had a higher incidence of toxicity.

**Vatalanib**: Vatalanib (PTK787/ZK 222584), an oral tyrosine inhibitor targeting VEGFR-1, VEGFR-2, VEGFR-3, PDGFR, c-kit, and c-fms, has been reported to have a potential role in advanced HCC [81]. In a phase I-II evaluation in combination with doxorubicin for treatment of patients with advanced HCC, the median PFS was 5.4 months with median OS of 7.3 months, and overall ORR was 26.0% in twenty-seven patient. There were 20% of patients with stable disease for at least 12 weeks. Grade 3 or 4 toxicities were mucositis (11%), alopecia (7%), and neutropenia (26%) [82]. The combination of vatalanib with doxorubicin seems to be promising for advanced HCC patients.

**Orantinib**: Orantinib (TSU-68) is an oral tyrosine kinase inhibitor of VEGFR-2, PDGFR, and FGFR which has some implication in the treatment of advanced HCC. In a phase I/II study of orantinib in patients with unresectable or metastatic HCC, there was an ORR of about 8.6% with the median TTP of 2.1 months and median OS of 13.1. The most common adverse events were hypoalbuminemia, gastrointestinal (diarrhea, anorexia, and abdominal pain), malaise, edema and AST/ALT elevation [83]. In combination with transarterial chemoembolization (TACE), a phase III study was conducted but was terminated after interim analysis did not reach the primary outcome of OS. The final analysis of 889 patients showed median OS of 31 months after TACE with TSU-68 and 32.3 months after TACE with placebo but the median time to TACE failure was longer in the TSU-68 arm with 23.9 months *vs.* 19.8 months for the placebo arm [84].

**Lenvatinib**: Lenvatinib is an orally inhibitor of multiple tyrosine kinase receptors including VEGFR1-3, FGFR1-4, PDGFR-alpha, RET protein and c-KIT [85]. It has been evaluated in a phase I/II study with 46 advanced HCC patients and demonstrated promising data with median OS of 18.7 months and median TTP of 12.8 months [86]. The ORR was 37% (no complete response) and stable disease was noted to be 47%. The most common adverse events were hypertension 76% (54% of grade 3), palmar-plantar erythrodysesthesia syndrome 61% (7%of grade 3), proteinuria 59% (20% of grade 3), anorexia 57% (2% of grade 3), thrombocytopenia 50% (33% of grade 3), and fatigue 48% (0% of grade 3). This led to a phase III randomized, open-label study evaluating lenvatinib *vs.* sorafenib to determine non-inferiority or superiority of lenvatinib [87]. The primary endpoint is OS. Secondary endpoints are PFS, TTR, ORR, safety, and PK/PD. This study has recently completed recruitment.

**Pazopanib**: Pazopanib is an oral multitarget kinase inhibitor targeting VEGFR, PDGFR, and c-Kit [88]. *In vitro* and *in vivo* studies showed inhibition of migration and invasion, induction of apoptosis of HCC cell lines, and significant reduction in tumor growth [89]. In a phase I study of pazopanib in hepatocellular carcinoma, twenty-eight patients were started on pazopanib for advanced hepatocellular carcinoma [90]. The most common adverse events were diarrhea, elevated liver enzyme, and skin hypopigmentation. Two out of twenty-eight patients had partial response and the median estimated PFS was 17.7 weeks [90].

**Regorafenib**: Regorafenib is multi-kinase inhibitor targeting angiogenesis (VEGFR1-3 and TIE2), tumor environment (PDGFR-beta and FGFR), and oncogenesis (KIT, RET, and RAF) [91]. The multikinase inhibitor has been evaluated in multiple solid tumors and has gained FDA approval for use in metastatic colorectal cancer patients who have failed standard therapy [92] and in patient with advanced gastrointestinal stromal tumors [93]. A phase II study was done to assess the safety of regorafenib in patient with HCC who progressed on sorafenib. The study had 36 patients and reported DCR of 72%, median TTP of 4.3 months, and median OS of 13.8 months. The most common adverse events were hand-foot syndrome, diarrhea, fatigue, hypertension, and hypothyroidism [94]. With the promising results from the phase II data, a phase III randomized, double-blind study of regorafenib in patient with HCC who progressed on sorafenib (RESORCE) is on-going.

### 5.2. Anti-VEGF/VEGFR Monoclonal Antibodies 

**Bevacizumab**: Bevacizumab is a humanized monoclonal antibody targeting VEGF causing direct antiangiogenic effects. Bevacizumab has been extensively studied in HCC as a single agent and in combination. In a phase II trial of bevacizumab in unresectable HCC, forty-six patients were treated with bevacizumab at 5 mg/kg or 10 mg/kg every 2 weeks. The study showed an ORR of 13% with median PFS of 6.9 months, and median OS of 12.4 months [95]. A similar phase II study done by Malk *et al.*, evaluated single agent bevacizumab at a dose of 5 to 10 mg/kg every 2 weeks in 30 patients. There were 3 patients with partial responses and 13 patients with stable disease [96]. Bevacizumab has also been evaluated in combination with other agents including gemcitabine, oxaliplatin, erlotinib, and capecitabine. In a phase II study of gemcitabine and oxaliplatin in combination with bevacizumab in patient with metastatic or unresectable HCC, the ORR was noted to be 20% with OS of 9.6 months and PFS of 5.3 months in 30 patients [97]. The major toxicities were leukopenia, neutropenia, hypertension, fatigue, and elevated liver enzymes [97]. Capecitabine with bevacizumab was evaluated by Hsu *et al.*, and noted some modest efficacy with ORR of 9%, median PFS and OS of 2.7 and 5.9 months, respectively [98]. Sun *et al.*, evaluated the combination of capecitabine and oxaliplatin with bevacizumab in a phase II study. Forty patients with unresectable HCC was enrolled in the study, and the median PFS was 6.8 months with OS of 9.8 months [99]. Both of these studies demonstrated relatively low toxicities and well tolerability with main side effects of peripheral neurotoxicity, fatigue, gastrointestinal symptoms, and hand-foot syndrome [98,99] (Table 2).

**Table 1:** Anti-VEGF TKIs.

Agent	Trial	Phase	Number	Patient	Therapy	Dosing	Results a *vs.* b
Sorafenib	Abou-Alfa *et al.* (2006) [51]	II	137	No prior systemic therapy	a. Sorafenib (*n* = 137)	Sorafenib 400 mg BID	OS: 9.2 months; TTP: 4.2 months; ORR: 8%; DCR: 41.6%
	Abdel-Rahman *et al.* (2013) [33]	II	52	No prior systemic therapy	a. Sorafenib (*n* = 26); b. Capecitabine (*n* = 26)	Sorafenib 400 mg BID; Capecitabine 1000 mg/m^2^ BID	OS: 7.05 months *vs.* 5.07 months, *p* < 0.016; PFS: 6 months *vs.* 4 months, *p* < 0.005; ORR: 15.5% *vs.* 3%
	SHARP (2008) [52]	III	602	No prior systemic therapy	a. Sorafenib (*n* = 299); b. Placebo (*n* = 303)	Sorafenib 400 mg BID	OS: 10.7 months vs 7.9 months, HR 0.69, *p* < 0.001; TTP: 4.1 months *vs.* 4.9 months, *p* = 0.77; ORR: 2% *vs.* 1%; DCR: 43% *vs.* 32%, *p* = 0.002
	Asia-Pacific (2009) [53]	III	226	No prior systemic therapy	a. Sorafenib (*n* = 150); b. Placebo (*n* = 76)	Sorafenib 400 mg BID	OS: 6.5 months *vs.* 4.2 months, HR 68, *p* = 0.014; TTP: 2.8 months *vs.* 1.4 months, HR 0.57, *p* = 0.0005; ORR: 3.3% *vs.* 1.3%; DCR: 53% *vs.* 12%, *p* = 0.0019
	Abou-Alfa *et al.* (2010) [54]	III	96	No prior systemic therapy	a. Sorafenib/Doxorubicin (*n* = 47); b. Doxorubicin/Placebo (*n* = 49)	Sorafenib 400 mg BID; Doxorubicin 60 mg/m^2^ q21days	OS: 13.7 months *vs.* 6.5 months, HR 0.49, *p* = 0.006; PFS: 6 months *vs.* 2.7 months, *p* = 0.006; TTP: 6.4 months *vs.* 2.8 months, HR 0.5, *p* = 0.02; ORR: 4% *vs.* 2%; DCR: 62% *vs.* 29%
	CALGB-80802; (NCT01015833)	III	R	No prior systemic therapy	a. Sorafenib/Doxorubicin; b. Sorafenib		Pending
	BOOST; (NCT01405573)	III	R	No prior anti-angiogenesis, Child-Pugh B only	a. Sorafenib; b. Placebo		Pending
Sunitinib	Zhu *et al.* (2009) [67]	II	34	No prior systemic therapy	a. Sunitinib (*n* = 34)	Sunitinib 50 mg daily, 4 weeks on, 2 weeks off	OS: 9.8 months; TTP: 4.1 months; ORR: 2.9%; DCR: 52.9%
	Faivre *et al.* (2009) [68]	II	37	No prior systemic therapy	a. Sunitinib (*n* = 37)	Sunitinib 50 mg daily, 4 weeks on, 2 weeks off	OS: 8.0 months; PFS: 3.7 months; TTP: 5.3 months; ORR: 2.7%; DCR: 37.8%
	SAKK 77/06 (2010) [66]	II	45	No prior systemic therapy	a. Sunitinib (*n* = 45)	Sunitinib 37.5 mg daily	OS: 9.3 months; PFS: 1.5 months; TTP: 1.5 months; ORR: 2%; DCR: 42%
	Barone *et al.* (2013) [65]	II	34	No prior systemic therapy	a. Sunitinib (*n* = 34)	Sunitinib 50 mg daily, 4 weeks on, 2 weeks off	OS: 5.8 months; TTP: 2.8 months; ORR: 11.8%; DCR: 44%
Linifanib	Toh *et al.* (2012) [72]	II	44	No prior systemic therapy	a. Llinifanib (*n* = 44)	Linifanib 0.25 mg/kg daily	OS: 9.7 months; TTP: 3.7 months; ORR: 9.1%
	Cainap *et al.* (2015) [73]	III	1035	No prior systemic therapy	a. Linifanib (*n* = 530); b. Sorafenib (*n* = 544)	Linifanib 17.5 mg daily; Sorafenib 400 mg BID	OS: 9.1 months *vs.* 9.8 months, HR 1.046; PFS: 4.2 months *vs.* 2.9 months, HR 0.813, *p* = 0.008; TTP: 5.4 months *vs.* 4.0 months, HR 0.895, *p* = 0.001; ORR: 10.1% *vs.* 6.1%
Brivanib	BRISK-FL (2013) [76]	III	1155	No prior systemic therapy	a. Brivanib (*n* = 577); b. Sorafenib (*n* = 578)	Brivanib 800 mg daily; Sorafenib 400 mg BID	OS: 9.5 months *vs.* 9.9 months, *p* = 0.3116; PFS: 4.2 months *vs.* 4.1 months, *p* = 0.8532); ORR: 12% *vs.* 9%, *p* = 0.0569; DCR: 66% *vs.* 65%, *p* = 0.8739
	BRISK-PS (2009) [77]	III	395	Failed sorafenib	a. Brivanib (*n* = 263); b. Placebo (*n* = 132)	Brivanib 800 mg daily	OS: 9.4 months *vs.* 8.2 months, HR 0.89, *p* = 0.3307; TTP: 4.2 months *vs.* 2.7 months, *p* < 0.001; ORR: 10% *vs.* 2%; DCR: 61% *vs.* 40%, *p* < 0.001
Cediranib	Alberts *et al.* (2012) [79]	II	28	No prior systemic therapy	a. Cediranib (*n* = 28)	Cediranib 45 mg daily	OS: 5.8 months; TTP: 2.8 months; DCR: 25%
	Zhu *et al.* (2013) [80]	II	17	Any line of therapy	a. Cediranib (*n* = 17)	Cediranib 30 mg daily	OS: 11.7 months; PFS: 5.3 months; DCR: 29%
Regorafenib	Bruix *et al.* (2013) [94]	II	36	Failed sorafenib	a. Regorafenib (*n* = 36)	Regorafenib 160 mg daily, 3 weeks on, 1 week off	OS: 13.8 months; TTP: 4.3 months; ORR: 3%; DCR: 72%
	RESORCE (NCT01774344)	III	R	Failed sorafenib	a. Regorafenib; b. Placebo	Regorafenib 160 mg daily, 3 weeks on, 1 week off	Pending
Orantinib	Kanai *et al.* (2011) [83]	I/II	35	Any line of therapy	a. Orantinib (*n* = 35)	Orantinib 400 mg BID	OS: 13.1 months; TTP: 2.1 months; ORR: 8.6%; DCR: 51.4%
Pazopanib	Yau *et al.* (2011) [90]	I	26	Prior therapy	a. Pazopanib (*n* = 26)	Pazopanib 200–800 mg daily	PFS: 17.7 weeks; ORR: 8%; DCR: 73%

OS: median overall survival; PFS: median progression free survival; TTP: median time to progression; ORR: objective response rate; DCR: disease control rate.

**Ramucirumab**: Ramucirumab is a human IgG1 monoclonal antibody that binds to the extracellular domain of VEGFR-2 to inhibit endothelial proliferation and migration [100]. In a phase I study to evaluate safety, maximum-tolerated dose (MTD), pharmacokinetics, and anticancer activity of ramucirumab, thirty-seven patients with advanced solid malignancies were given ramucirumab and 30% of the patients were found to have partial response or stable disease for at least 6 months with dose limiting toxicities of hypertension and deep vein thrombosis [101]. In a phase II evaluation of ramucirumab as first line monotherapy for advanced hepatocellular carcinoma, forty-two patients received ramucirumab at 8 mg/kg every two weeks and showed a median PFS of 4.0 months, ORR of 9.5%, and median OS of 12.0 months [100]. The most common grade 3 or more toxicities were hypertension (14%), gastrointestinal bleeding (7%), infusion-related reaction (7%), and fatigue (5%) [100]. Subsequently, a phase III trial (REACH) was conducted in patients with advanced HCC. The study compared ramucirumab with supportive care to placebo with supportive care in second-line treatment after sorafenib. The results suggested that there might be some benefit of ramucirumab in the sub-population of HCC patients with AFP greater than ≥400 ng/mL or ≥1.5 times the upper limits of normal [102,103] (Table 2).

**Table 2:** Anti-VEGF/VEGFR Antibodies.

Agent	Trial	Phase	Number	Patient	Therapy	Dosing	Results a *vs.* b
Bevacizumab	Malka *et al.* (2007) [96]	II	30	Prior therapy	a. Bevacizumab (*n* = 30)	Bevacizumab 5 or 10 mg/kg every 2 weeks	DCR: 67%
	Siegel *et al.* (2008) [95]	II	46	Maximum 1 prior line of therapy	a. Bevacizumab (*n* = 46)	Bevacizumab 5 or 10 mg/kg every 2 weeks	OS: 12.4 months; PFS: 6.9 months; ORR: 13% (8.3% for 5 mg/kg;14.7% for 10 mg/kg)
	NCT00881751	II		No prior systemic therapy	a. Bevacizumab with erlotinib; b. Sorafenib		Pending
Ramucirumab	Zhu *et al.* (2013) [100]	II	42	No prior systemic therapy	a. Ramucirumab (*n* = 42)	Ramucirumab 8 mg/kg every 2 weeks	OS: 12.0 months; PFS: 4.0 months; TTP: 4.2 months; ORR: 9.5%; DCR: 69.0%
	REACH (2015) [102,103]	III	565	Failed sorafenib	a. Ramucirumab (*n* = 283); b. Placebo (*n* = 282)	Ramucirumab 8 mg/kg every 2 weeks	OS: 9.2 months *vs.* 7.6 months, HR 0.87, *p* = 0.14; PFS: 2.8 months vs 2.1 months, HR 0.63, *p* ≤ 0.0001; TTP: 3.5 months *vs.* 2.6 months, HR 0.59, *p* ≤ 0.0001; ORR: 7% *vs.*<1%; DCR: 56% *vs.* 46%

OS: median overall survival; PFS: median progression free survival; TTP: median time to progression; ORR: objective response rate; DCR: disease control rate.

### 5.3. Epidermal Growth Factor Receptor (EGFR)

Epidermal growth factor receptor (EGFR) pathway plays an important role in liver injury and the pathogenesis of HCC [104,105,106]. EGFR has been noted to be frequently overexpressed in HCC [107]. Because of the promising data, there have been efforts to evaluate the efficacy of targeting the EGFR pathway as a potential therapeutic option for HCC (Table 3).

**Cetuximab**: Cetuximab is a recombinant IgG1 monoclonal antibody that competitively inhibits ligand bindings through binding of the extracellular domain of the EGFR. The potential use of cetuximab in advanced HCC was evaluated in two phase II studies. Both did not show any responses to cetuximab [108,109]. Cetuximab in combination with gemcitabine and oxaliplatin was evaluated in phase II studies which demonstrated a modest response rate of 12.5%–20% with median TTP of 4.5–4.7 months and OS of 4.4–9.5 months [110,111]. The toxicities were generally manageable but there are concerns over the lower than expected TTP and OS as compared to historical sorafenib.

**Erlotinib**: Erlotinib is an oral selective tyrosine kinase inhibitor of EGFR by targeting the binding of ATP to the tyrosine kinase domain of EGFR [112]. Huether and colleagues demonstrated the inhibition of EGRFR enzyme by erlotinib which results in growth inhibition, cell cycle arrest, and apoptosis of human HCC cell lines [113]. In a phase II study of 38 patients with HCC treated with erlotinib, Philip *et al.*, reported an ORR of 9%, 32% were progression free at six month, disease control rate of 59%, and median OS of 13 months [114]. In another phase II study by Thomas *et al.*, 40 patients with unresectable HCC were treated with erlotinib and reported an OS of 10.75 months with 43% PFS at 16 weeks [115]. In the phase III SEARCH clinical trial, 720 patients with advanced HCC and Child-Pugh class A cirrhosis were randomized to treatment with sorafenib plus either erlotinib or placebo [115]. Erlotinib did not improve survival when added to sorafenib with the median OS of 9.5 months *vs.* 8.5 months (HR, 0.929; 95% CI, 0.781–1.106; *p* = 0.408) [116]. The time to progression was 3.2 months *vs.* 4.0 months (HR, 1.135; 95% CI, 0.944–1.366; *p* = 0.18. The ORR showed a trend toward sorafenib with erlotinib at 6.6% *vs.* 3.9% [116]. The toxicities between the two arms were comparable but there were higher rates of diarrhea, rash, and anorexia in the group receiving sorafenib and erlotinib [116]. In the combination of erlotinib and bevacizumab, multiple phase II trials have demonstrated modest efficacy with ORR 0%–24%, TTP 1.81–3 months, and median OS 4.37–13.7 months [117,118,119,120,121]. The common toxicities from the studies are hypertension, fatigue, rash, and diarrhea. The combination of docetaxel with erlotinib did not show any advantages [122].

**Lapatinib**: Lapatinib is a dual inhibitor of EGFR and HER2/NEU tyrosine kinases which have had some activity in HCC. In two phase II studies, lapatinib was evaluated in patients with advanced HCC and showed an ORR of 5%, median PFS of 1.9–2.3 months, and median OS of 6.2–12.6 months [123,124].

**Vandetanib**: Vandetanib is an oral inhibitor of VEGFR and EGFR signaling pathways which have been shown to have significant PFS benefit in patient with advanced medullary thyroid cancer and advanced non-small cell lung cancer. Due to the known anti-tumor activities of VEGFR and EGFR inhibitors in advanced HCC, vandetanib was evaluated in a phase II study but did not show any statistically significant in PFS or OS as compared to placebo [125].

### 5.4. Mechanistic (Mammalian) Target of Rapamycin (mTOR)

The mTOR is a serine/threonine kinase in the PI3K/Akt pathway which plays an important role in angiogenesis and cell proliferation. mTOR upregulation has been reported in 5%–15% of HCC and increase expression of P70 S6 kinase (downstream of mTOR) in 45% of HCC [126]. The increase in expression of mTOR and its downstream kinase made this pathway an attractive target for potential therapeutic intervention. Huynh *et al.*, evaluated the effect of everolimus, an mTOR inhibitor, in patient-derived HCC xenografts [127]. They reported inactivation of downstream target of mTOR, reduced VEGF expression, cell proliferation inhibition, and down-regulation of key cell transcription factors including cyclin B1, c-Myc, Cdk-4, Cdk-2, and cdc-2 [127]. Currently, there are multiple mTOR inhibitors available and have been evaluated in HCC including everolimus (Table 4), temsirolimus, and sirolimus [128,129,130,131,132,133].

**Table 3:** EGFR Targets.

Agent	Trial	Phase	Number	Patient	Therapy	Dosing	Results a *vs.* b
Cetuximab	Gruenwald *et al.* (2007) [109]	II	27	Prior therapy	a. Cetuximab (*n* = 27)	Cetuximab 400 mg/m^2^ follow by weekly 250 mg/m^2^	TTP: 8.0 weeks; DCR: 44.4%
	Zhu *et al.* (2007) [108]	II	30	Prior therapy	a. Cetuximab (*n* = 30)	Cetuximab 400 mg/m^2^ follow by weekly 250 mg/m^2^	OS: 9.6 months; PFS: 1.4 months; TTP: 4.2 months; DCR: 17%
Erlotinib	Philip *et al.* (2005) [114]	II	38	Maximum 1 prior line of therapy	a. Erlotinib (*n* = 38)	Erlotinib 150 mg daily	OS: 13 months; TTP: 3.8 months; ORR: 9%; DCR:50%
	Thomas *et al.* (2007) [115]	II	40	No prior systemic therapy	a. Erlotinib (*n* = 40)	Erlotinib 150 mg daily	OS: 10.75 months; PFS: 43% at 16 weeks; TTP: 13.3 weeks; DCR: 43%
	Zhu *et al.* (2015) [116]	III	720	No prior systemic therapy	a. Sorafenib/Erlotinib (*n* = 362); b. Placebo (*n* = 358)	Sorafenib 400 mg BID; Erlotinib 150 mg daily	OS: 9.5 months *vs.* 8.5 months, HR 0.929, *p* = 0.408; TTP: 3.2 *vs.* 4.0 months, HR 1.135, *p* = 0.18; ORR: 6.6% *vs.* 3.9%, *p* = 0.102; DCR: 52.5% *vs.* 43.9%, *p* = 0.021
	Govindarajan *et al.* (2013) [117]	II	21	No prior systemic therapy	a. Bevacizumab/Erlotinib (*n* = 21)	Bevacizumab 15 mg/kg every 3 weeks; Erlotinib 150 mg daily	OS: 8.33 months; PFS: 28% at 27 weeks; TTP: 2.57 months
	Yau *et al.* (2012) [118] (study halted)	II	10	Failed sorafenib	a. Bevacizumab/Erlotinib (*n* = 10)	Bevacizumab 10 mg/kg every 2 weeks; Erlotinib 150 mg daily	OS: 4.37 months; TTP: 1.81 months; DCR: 0%
	Hsu *et al.* (2013) [119]	II	51	No prior systemic therapy	a. Bevacizumab/Erlotinib (*n* = 51)	Bevacizumab 5 mg/kg every 2 weeks; Erlotinib 150 mg daily	OS: 10.7 months; PFS: 2.9 months; ORR: 6%; DCR: 53%
	Philip *et al.* (2012) [120]	II	27	Maximum 1 prior line of therapy	a. Bevacizumab/Erlotinib (*n* = 27)	Bevacizumab 10 mg/kg every 2 weeks; Erlotinib 150 mg daily	OS 9.5 months; TTP: 3.0 months; ORR: 5%
	Kaseb *et al.* (2012) [121]	II	59	Maximum 1 prior line of therapy	a. Bevacizumab/Erlotinib (*n* = 59)	Bevacizumab 10 mg/kg every 2 weeks; Erlotinib 150 mg daily	OS: 13.7 months; PFS: 7.2 months; ORR: 24%; DCR: 80%
Lapatinib	Bekaii-Saab *et al.* (2009) [123]	II	26	Maximum 1 prior line of therapy	a. Lapatinib (*n* = 26)	Lapatinib 1500 mg daily	OS: 12.6 months; PFS 1.9 months; ORR: 40%
	Ramanathan *et al.* (2009) [124]	II	40	Maximum 1 prior line of therapy	a. Lapatinib (*n* = 40)	Lapatinib 1500 mg daily	OS: 6.2 months; PFS: 2.3 months; ORR: 5%; DCR: 40%

OS: median overall survival; PFS: median progression free survival; TTP: median time to progression; ORR: objective response rate, DCR: disease control rate.

**Table 4:** mTOR Target.

Agent	Trial	Phase	Number	Patient	Therapy	Dosing	Results a vs b
Everolimus	Zhu *et al.* (2011) [128]	I/II	28	Prior therapy	a. Everolimus (*n* = 28)	Everolimus 5 or 10 mg daily	OS: 8.4 months; PFS: 3.8 months; ORR: 4%; DCR: 44%
	EVOLVE-1 (2014) [129]	III	546	Failed sorafenib	a. Everolimus (*n* = 362); b. Placebo (*n* = 184)	Everolimus 7.5 mg daily	OS: 7.6 months *vs.* 7.3 months; TTP: 3.0 months *vs.* 2.6 months, HR 0.93 DCR: 56.1% *vs.* 45.1%, *p* = 0.01

OS: median overall survival; PFS: median progression free survival; TTP: median time to progression; ORR: objective response rate; DCR: disease control rate.

**Everolimus**: One of the most well studied mTOR inhibitors is everolimus. In a study to determine maximum tolerated dose (MTD) of everolimus in advanced HCC patients, Shiah *et al.*, identified the MTD as 7.5 mg daily and 70 mg weekly. The efficacy data demonstrated DCR of 71.4% *vs.* 44.4%, median PFS was 3.7 months *vs.* 1.9 months, and median OS of 7.7 months *vs.* 5.7 months for daily and weekly dose respectively in 39 patients with advanced HCC [130]. In a phase I/II study of everolimus for 28 patients with advanced HCC, Zhu and colleagues reported an ORR of 4%, median PFS of 3.8 months, and median OS of 8.4 months [128]. Everolimus was assessed in the EVOLVE-1 trial which is a randomized, double-blind, phase III study with 546 patients with advanced HCC who progressed on sorafenib or was intolerant of sorafenib. The study reported no significant differences in overall survival between the everolimus and placebo group. The median OS was 7.6 months *vs.* 7.3 months, median TTP was 3.0 months *vs.* 2.6 months, and DCR was 56.1% and 45.1% (*p* = 0.01) for everolimus *vs.* placebo respectively [129]. The most common side effects were anemia, asthenia, and decreased appetite. In a currently ongoing phase II trial of everolimus in combination with sorafenib *vs.* sorafenib alone for first line therapy in 106 (93 evaluable for primary endpoint) advanced HCC, Koeberle and colleagues reported the ORR 0% *vs.* 10%, median PFS was 6.6 months *vs.* 5.7 months, median TTP was 7.6 months *vs.* 6.3 months, and median OS 10 months *vs.* 12 months in the sorafenib alone *vs.* everolimus with sorafenib combination [131]. It is unlikely there would be any large randomized study for testing the combination.

### 5.5. MET-HGF

MET receptor tyrosine kinase has been noted to play an important role in tumorigenesis. Hepatocyte growth factor binds to MET tyrosine kinase and activates the downstream RAS-MAPK and PI3K-AKT pathways which result in cell proliferation, blood vessel formation, and cellular migration [134]. 

**Tivantinib** (ARQ 197) is an oral inhibitor of c-MET and has showed some promising data in advanced HCC. A randomized, placebo-controlled phase II study of tivantinib for second-line treatment of advanced HCC was performed to assess for efficacy and safety [135]. Seventy-one patients were randomized to receive tivantinib and 36 patients were randomized to the placebo group. The median TTP was 1.6 months for patients treated with tivantinib and 1.4 months for the placebo group (HR 0.64; 90% CI, 0.43–0.94; *p* = 0.04). The study also demonstrated patients with MET-high tumor had median TTP were longer in the tivantinib group (2.7 months) *vs.* 1.4 months in the placebo group (HR 0.43; 95% CI, 0.19–0.97; *p* = 0.03). The most common grade 3 or higher adverse events in the tivantinib group were neutropenia and anemia [135]. 

**Cabozantinib** (XL184) is another oral inhibitor of MET and VEGFR2 currently showing potential antitumor activity in HCC. In a phase II study of the activity of cabozantinib, 41 patients were treated with cabozantinib for 12 weeks and evaluated in a randomized discontinuation method. Patient with partial response at 12 weeks were continued on cabozantinib, patient with stable disease were randomized to placebo or continuation of cabozantinib, and patients with progression of disease were discontinued. At week 12, the DCR was noted to be 68% [136]. Tivantinib and cabozantinib are currently being evaluated in phase III trials. 

### 5.6. Immune Checkpoint Blockage

Immunotherapy has been one of the most recently exciting areas in cancer therapy. Immune checkpoint inhibitors are currently the primary agents used in anti-tumor immunity and have been shown to be effect in advanced melanoma and lung cancer. Cytotoxic T lymphocyte associated antigen 4 (CTLA-4) and programmed cell death protein 1 (PD1) are two major targets of these immune checkpoint inhibitors agents [137]. Targeting the immune system is a promising therapeutic option especially in HCC. One of the hypotheses to the pathogenesis of HCC suggests the development of tumor cell in an environment which is persistently activated by inflammatory factors and cytokines [138]. The microenvironment of the liver has a major role in the evasion of HCC from the immune system. There are multiple immunosuppressive mechanisms which have been suggested to explain this evasion of the immune system including suppression of natural killer cells, defective antigen presentation, recruitment of T regulatory and myeloid-derived suppressor cells which suppress immune response, and overexpression of immune checkpoint pathways such as CTLA-4 and programmed cell death protein 1 ligand (PD-L1) [138]. Activation of the immune system has been noted in local ablative therapies such as TACE and RFA can stimulate infiltration of tumor specific T-cell, activation of natural killer cells, and increase release of cytokines causing tumor regression [137,138].

**CTLA-4**: CTLA-4 is expressed on T regulatory cells and active T cell which subsequently suppress immune responses by inhibiting binding of antigen presentation by antigen-presenting cell and T-cell activation. Tremelimumab, a monoclonal antibody which blocks CTLA-4, has been evaluate in patient with chronic hepatitis C and HCC in a phase I study demonstrating DCR of 76.4% and median TTP of 6.48 months [139]. The safety profile was well tolerated without any immune related toxicity requiring steroid. Another phase I trial of Tremelimumab with chemoembolization or RFA is currently recruiting patients (NCT01853618). This is promising given data showing immune response stimulation after TACE and RFA.

**PD-1**: PD-1 is a part of the CD28 superfamily which helps in TCR receptor signaling to stimulate T regulatory and myeloid-derived suppressor cells. In normal condition, the binding of PD1 to its ligands (PD-L1 or PD-L2) induces T regulatory cell proliferation and differentiation and inhibition of T-cell proliferation. Tumor cells are able to utilize the PD1/PD-L1/PD-L2 to evade the immune system. PD-L1 and PD1 have been demonstrated in HCC tumor cells and T cells in tumor tissue specimens [138]. At the 2015 American Society of Clinical Oncology (ASCO) annual meeting, preliminary data on nivolumab was presented [140]. Nivolumab, a fully human IgG4 monoclonal antibody PD-1 inhibitor, has shown excellent results in unresectable or metastatic melanoma and metastatic non-small cell lung cancer. Preliminary report was on the phase I/II study of nivolumab in 47 patients with advance HCC Child Pugh class A or B who progressed on sorafenib, refused sorafenib, or intolerant of sorafenib [140]. The primary endpoint was safety with secondary points as antitumor activity using mRECIST criteria, pharmacokinetics, and immunogenicity. Response was evaluated in 42 patients and noted the OS at 12 months was 62%, ORR was 19%, and DCR of 67%. The most common adverse events were rash, elevate liver enzymes, elevated lipase, and elevated amylase [140]. Although the study is relative small, however, the results are very promising. Therefore, many larger trials with immune checkpoint inhibitors are planning and ready to go for the further evaluation.

## 6. Future Directions

Hepatocellular carcinoma is one of the most common malignancies worldwide with the worst morbidity and mortality. Even with the landmark development of sorafenib which advances survival for patients with advanced HCC, the overall outcome of HCC is still disappointing, and we have not made much advancement in other targeted therapeutics. Numerous agents suggested some encouraging data in pre-clinical models and early phase clinical trials but failed to show significant clinical benefits in later and larger randomized studies. The lack of effective targeted therapeutics may be related to our limited understanding of the pathogenesis of HCC in general. The mechanisms behind hepatocarcinogenesis and progression of disease are complex and heterogeneous. The behavior and underlying biology of HCC is likely related to the etiological factors which influence the HCC development in distinct processes. Each etiological factor will likely drive a different genomic alteration and pathway. Our understanding of these alternations is limited at the moment. With more understanding of the mechanism of hepatocarcinogenesis, we may be able to develop more precise specific mechanism based and oncogenic pathways target studies. In particularly, immunotherapy is likely the area that may bring outbreaks in HCC treatment in the future. Initial evaluations into immunotherapy for HCC focused mainly on vaccination. These approaches demonstrated promising preclinical data but lacked benefit in clinical trials [141]. The poor results in clinical trial may be secondary to the redundant system of immune regulation of HCC including ability to prime naïve T cells without costimulations, lower natural killer and dendritic cells frequencies, T regulatory cells accumulation, and increased PD-1-B7-H1 interaction which leads to immune tolerance [142]. With recent understanding of checkpoint inhibition in melanoma and lung, there has been more interest in evaluating these agents in HCC. Inhibition of these regulatory mechanisms in HCC with new checkpoint inhibitors is one of the new promising directions of molecular targeted therapeutics in HCC. These molecularly targeted therapeutics are usually not metabolism in the liver which would not be limited by underlying liver disease. Although promising, there are also limitations to checkpoint inhibition including lack of immune response in some patients, development of tumor evasion of previous immune response, and lack of response in certain tumor types. This may allow for more novel combination therapy including immunotherapeutic with small molecules, chemotherapy, and local therapy such as TACE or RFA. These are exciting new areas which have yet to be fully developed.
